# A Weighted-LSM Method to Improve Classification and Concentration Evaluation from Laser-Induced Fluorescence Spectra

**DOI:** 10.3390/s22207721

**Published:** 2022-10-11

**Authors:** Valentina Gabbarini, Alessandro Puleio, Riccardo Rossi, Andrea Malizia, Pasqualino Gaudio

**Affiliations:** 1 Department Industrial Engineering, University of Rome Tor Vergata, Via del Politecnico, 1, 00133 Rome, Italy; 2 Department of Biomedicine and Prevention, University of Rome Tor Vergata, Via Montpellier, 1, 00133 Rome, Italy

**Keywords:** laser-induced fluorescence, biological agents, detection, optical techniques, least square minimization method

## Abstract

The detection of biological agents using optical systems is an open field of research. Currently, different spectroscopic techniques allow to detect and classify chemical agents while a fast and accurate technique able to identify biological agents is still under investigation. Some optical techniques, such as Laser-Induced Breakdown Spectroscopy (LIBS) or Laser-Induced Fluorescence (LIF), are already used as classification methods. However, the presence of background, spectrum similarities and other confounders make these techniques not very specific. This work shows a new method to achieve better performances in terms of classification and concentration evaluations. The method is based on the Weighted Least Square Minimization method. In fact, by using ad hoc weights, the LSM looks at specific features of the spectra, resulting in higher accuracy. In order to make a systematic analysis, numerical tests have been conducted. With these tests, the authors were able to highlight the various advantages and drawbacks of the new methodology proposed. Then, the method was applied to some LIF measurements to investigate the applicability of the method to preliminary experimental cases. The results show that, by using this new weighted LSM, it is possible to achieve better classification and concentration evaluation performances. Finally, the possible application of the new method is discussed.

## 1. Introduction

Laser-Induced Breakdown Spectroscopy (LIBS) technique, Absorption-based techniques and Laser-Induced Fluorescence (LIF) technique can be considered among the most known spectroscopic methods for spectra analysis.

To extrapolate the proper pieces of information from the spectra obtained through these methods, complex and ad hoc algorithms must be usually applied. In many applications, the study of the spectra may require machine learning techniques, since the interactions at the basis of the spectra may be different and hard to explain.

The main goal of this work is to discuss how the Least Square Minimization method (LSM) could be improved when applied to fluorescence spectra of bio-agents obtained by means of LIF. 

The fluorescence phenomenon is a type of electromagnetic emission derived from an electromagnetically excited state molecule [1]. To date, fluorescence is the basis of dominant methodologies widely used in biotechnology and biological analyses [1,2,3,4,5]. A variety of molecules, known as fluorophores, emit fluorescence. 

The LIF is one of the best candidates for developing optical devices able to detect, classify and measure the concentration of biological agents in the environment, due to the presence of fluorophores in each one. A different fluorophores composition results in a different LIF spectrum as a fingerprint of the bio-agent itself. Already applied for sensors and stand-off techniques, its capabilities for classification are quite limited. The poor classification performances are mainly due to the enormous variability of organic substances in the environment that can emit fluorescent light and to the high spectra similarity among similar biological agents. 

LIF classification accuracy can be increased by both hardware and software implementation. First of all, by exciting the bio-agents with more wavelengths, it is possible to obtain more spectra and thus more useful information for proper classification. Then, the development of a more sophisticated algorithm allows the increase in accuracy and reduces the false negative rate. In recent years, several algorithms have been investigated, such as Principal Component Analysis (PCA), Support Vector Machine (SVM) and Decision Tree [6,7,8,9].

This work aims at introducing a new Weighted Least Square Minimization (W-LSM) method to evaluate the concentration of specific fluorescent agents in a mixture sample and then classify them. This new method allows the increase in the performance of LSM in tricky situations, where the reference spectra of the fluorescent agents are similar and their classification is awkward.

The paper is organized as follows: The next section introduces the theory of the method and all the new parameters used in this work to describe the results. Section 3 shows a series of numerical cases that have been used to investigate the capabilities of this new method. The use of synthetic cases allows the performance of a benchmark of the various cases, highlighting the advantages and limits of the proposed method. In Section 4, some preliminary experimental tests have been performed to validate the applicability of the technique. Section 5 summarizes the findings and draws the conclusions of this work.

## 2. Theory of the Method

### 2.1. Laser-Induced Fluorescence 

The fluorescence phenomenon takes origin from specific molecules, in which the electrons of the outer ring, which are normally presented in a ground state of energy (S_0_), jump to an excited state (S_1_) due to the energy received from an electromagnetic wave [1,10]. The following return to the ground state of electrons takes place with the emission of energy in form of the electromagnetic wave as luminescence [1]. In fluorescence phenomena, the processes that take place during the absorption and the following emission of light can be represented by the Jablonsky diagram [1]. 

It is important to highlight that the fluorescence light is proportional to the absorbed light, according to the Lambert–Beer law [1]:

(1) $$I_{F}\propto I_{A}=I_{0}-I_{t}=I_{0}\left(1-e^{-\sigma cd}\right)$$

where $I_{F}$ is the fluorescence light intensity; $I_{A}$ is the absorbed light; $I_{0}$ and $I_{A}$ are, respectively, the incident light used to excite the sample and the transmitted light; *σ* is the absorption cross-section of the agents; *c* is the concentration; and *d* is the optical path. Since the product σcd is usually small, the exponential can be simplified by its Taylor series (first order), and a linear correlation between fluorescence and concentration can be found:

(2) $$I_{F}\propto I_{A}=I_{0}-I_{t}=I_{0}\sigma cd$$

It is common to express the link between fluorescence and absorbed light using a parameter (*η*) that represents the ratio between the two intensities ($I_{F}=\eta I_{A}$).

### 2.2. Classical Least Square Minimisation Method (C-LSM)

The Least Square Minimization method (LSM) is a method for regression analysis used in the statistical and other fields. In particular, this method is used to find the best fit among data. It is useful in sets of equations where the equations are more than the unknowns. In particular, the method finds the best set of unknowns that minimizes the “least squares” [11].

In this work, the authors used this method applied to the LIF spectra analyses. If it is considered a sample that contains a number *m* of biological agents, the fluorescence spectrum emitted from this sample is a result of the induced fluorescence emission of each biological agent present in it. Thus, this work used the mathematical method of LSM to compare the mixture sample spectrum with a database containing the LIF spectrum of each biological agent. Through this method, it may be possible to detect, identify (or classify) and measure the concentration of each biological agent in the mixture sample.

The acquisition of the spectrum was carried out through a spectrometer and the spectrum had a number *N* of measured wavelengths (indicated as *λ*). The sample mixture contained unknown agents with unknown concentrations. Thus, the start hypothesis was to suppose a linear dependence between the intensity of the spectrum (*I*) and the concentration (*c*) of the bio-agents sample:

(3) $$I_{final,m}\left(\lambda_{i}\right)=\sum_{j=1}^{m}I_{j}\left(\lambda_{i}\right)c_{j}$$

where the index *j* is for the *j*-th agent and *i* to the *i*-th wavelength. From the previous equation, a system of equations with *m* unknown factors, which represent the concentration of each biological agent, and *n* equations (equal to the number of measured wavelengths named *N*) was obtained.

(4) $$\left\{\begin{array}{c} I_{final}\left(\lambda_{1}\right)=c_{1}I_{1}\left(\lambda_{1}\right)+c_{2}I_{2}\left(\lambda_{1}\right){+}_{\ldots \ldots }+c_{m}I_{m}\left(\lambda_{1}\right)+\varepsilon_{1}\\ I_{final}\left(\lambda_{2}\right)=c_{1}I_{1}\left(\lambda_{2}\right)+c_{2}I_{2}\left(\lambda_{2}\right){+}_{\ldots \ldots }+c_{m}I_{m}\left(\lambda_{2}\right)+\varepsilon_{2}\\ \begin{array}{c} \vdots \\ I_{final}\left(\lambda_{n}\right)=c_{1}I_{1}\left(\lambda_{n}\right)+c_{2}I_{2}\left(\lambda_{n}\right){+}_{\ldots \ldots }+c_{m}I_{m}\left(\lambda_{n}\right)+\varepsilon_{n} \end{array} \end{array}\right.$$

where $\varepsilon_{i}$ represents the error (due to noise, etc.) associated with the count measurement at the wavelength $\lambda_{i}$. Expressing the system in matrix form, it follows:

(5) Σc=I−E

where

(6) $$\Sigma =\left[\begin{array}{ccc} I_{1}\left(\lambda_{1}\right) & \ldots & I_{m}\left(\lambda_{1}\right)\\ \vdots & \ddots & \vdots \\ I_{1}\left(\lambda_{n}\right) & \cdots & I_{m}\left(\lambda_{n}\right) \end{array}\right];\;c=\left[\begin{array}{c} c_{1}\\ \vdots \\ c_{m} \end{array}\right];\;I=\left[\begin{array}{c} I_{tot}\left(\lambda_{1}\right)\\ \vdots \\ I_{tot}\left(\lambda_{n}\right) \end{array}\right];E=\left[\begin{array}{c} \varepsilon_{1}\\ \vdots \\ \varepsilon_{n} \end{array}\right]$$

The LSM involves that the square of the error is minimized, which means that $E^{T}E=min$. Thus, we can write:

(7) $$\frac{\partial {Ε}^{T}E}{\partial c}=2\frac{\partial E^{T}}{\partial c}E=2\Sigma^{T}\left(\Sigma c-I\right)=0$$

The previous equation solution allows the calculation of the best concentration vector with the minimization of the error, as follows:

(8) $$c={\left(\Sigma^{T}\Sigma \right)}^{-1}\Sigma^{T}I$$

Thus, an algorithm to calculate the concentration of biological agents in a mixture sample was developed. From now on, this method, which is the standard or classical one, will be called Classical-LSM (or C-LSM). In order to correctly work, it is fundamental that the number of unknown agents (*m*) is smaller than the number of measured wavelengths (*n*).

### 2.3. Weighted LSM Method Based on Feature Differences (W_DIF_-LSM)

The C-LSM method places the same importance on each intensity, independently of the wavelength. However, in some conditions, some wavelengths can have different importance if compared with other ones. In general, it is expected that the method should look at those regions where the features of different agents differ strongly. Therefore, the LSM was updated by introducing a weighting matrix that weighs more the regions that should be more agent-discriminant. 

Starting from the unweighted LSM, where $E^{T}E=min$, it is possible to introduce a weight matrix W, such that $E^{T}WE=min$. For example, if the *i*-th element of the matrix W is zero, this measurement will not be considered, while if it is a large value (respect with other elements), this element will strongly be considered. In this work, a diagonal W matrix was considered:

(9) $$W=\left[\begin{array}{ccc} W_{1} & \cdots & 0\\ \vdots & \ddots & \vdots \\ 0 & \cdots & W_{n} \end{array}\right]$$

Additionally, by following the same steps of the previous section, Equation (7) becomes:

(10) $$\frac{\partial {Ε}^{T}WE}{\partial c}=\frac{\partial E^{T}}{\partial c}WE+E^{T}\frac{\partial W}{\partial c}E+E^{T}W\frac{\partial E}{\partial c}=0$$

The term “$E^{T}\frac{\partial W}{\partial c}E$” is zero if the weights are independent by the concentration. This is the first approximation. However, it is worth mentioning that, in the case of non-linearity (see Equations (1) and (2)), a concentration-dependent weight may be introduced to increase the performance. Thus, Equation (5) becomes:

(11) $$\Sigma^{T}W\left(\Sigma c-I\right)+\left(\Sigma^{T}c^{T}-I\right)\Sigma =0$$

Additionally, by resolving the system, it can be found:

(12) $$c={\left(\Sigma^{T}W\Sigma \right)}^{-1}\left(\Sigma^{T}WI\right)$$

Of course, a relevant role is played by how the weight matrix is defined. In this work, the weight matrix was defined as the relative difference among the spectra of the different agents:

(13) $$W_{dif,i}=\frac{\left|I_{d,1_{i}}-I_{d,2_{i}}\right|}{\max\left(I_{d,1_{i}}+I_{d,2_{i}},1\right)}$$

where $I_{d,1,i}$ and $I_{d,2,i}$ are the normalised values of the spectra databases of the two agents. The maximum function at the denominator was used to avoid infinity values due to numerical issues (0/0). 

Furthermore, another term must be introduced to determine the weights matrix, parameter *A*, which regularizes the different weight matrix respect with the identity matrix. Thus, Equation (13) becomes:

(14) $${\bar{W}}_{dif_{A}}={\bar{W}}_{dif}+\bar{Id}*mean\left(\bar{W}\right)*A$$

If the value *A* is not considered, the algorithm places importance only on the wavelengths that have a consistent difference. This effect may reduce the information used by the LSM excessively, to a degree that the weights have an opposite effect, reducing the accuracy of the algorithm. 

Finally, the concentration was calculated as follows:

(15) $$c={\left(\Sigma^{T}W_{dif_{A}}\Sigma \right)}^{-1}\left(\Sigma^{T}W_{dif_{A}}I\right)$$

## 3. Numerical Analyses

At first, the performances of the new algorithm were analyzed and compared with the classic one by numerical simulations. The numerical analyses aimed to identify limits and to improve the algorithms proposed. In order to perform numerical simulations on the two LSM algorithms, software with the capability to create (or simulate) a synthetic spectrum obtained from two synthetic spectra and agent concentration was developed. Each synthetic spectrum is a function of the concentration values of simulated Biological Agents (Bas). Briefly, the numerical tests performed can be described as follows:

1. Database spectra generation by analytical equations;
2. Generation of the “measured” spectrum;
3. Noise addition to simulate a real spectrum;
4. Spectrum analysis with C-LSM and W_DIF_-LSM.

### 3.1. Method

The software routine was programmed using LabView software (National Instruments Corp, Austin, TX, USA) [12]. All the numerical tests were conducted supposing only two biological agents. The simulated spectra were generated by the following equation:

(16) $$I_{j}\left(\lambda_{i}\right)=\frac{\lambda_{i}-\lambda_{s}}{\sigma^{2}}e^{-\frac{{\left(\lambda_{i}-\lambda_{s}\right)}^{2}}{2\sigma^{2}}}$$

where the $\lambda_{s}$ and *σ* are free constants, with which it is possible to simulate different spectrum shapes. In addition to the previous equation, another equation was used to add peaks in the spectrum:

(17) $$I_{p}{}_{j}=I\;\left[\frac{\gamma^{2}}{{\left(\lambda_{i}-\lambda_{0}\right)}^{2}+\gamma^{2}}\right]$$

where the *I*, $\lambda_{0}$ and *γ* are three free constants that indicate, respectively, the maximum intensity, the position and the amplitude of the second peak. The two simulated spectra created were used from the software to generate the database (*Σ*). 

After the generation of the two synthetic spectra, two different concentrations were imposed to simulate the presence of two simulated biological agents through Equation (3). The software composes the final synthetic spectrum in order to simulate a hypothetical mixture sample of biological agents.

In addition, to simulate real conditions, a Gaussian random noise signal was added to simulate spectra as described in Equation (18):

(18) $$I_{noisy,j}\left(\lambda_{i}\right)=I_{j}\left(\lambda_{i}\right)\left(N*\left(random\left(-1,1\right)+1\right)\right)$$

where the variable *N* indicates a free percentage value of noise for the spectrum.

Finally, the C-LSM and W_DIF_-LSM algorithms were applied to the spectrum.

The residuals were calculated as the difference between the “measured” concentration ($c_{1,mes}$ and $c_{2,mes}$) and the imposed concentrations ($c_{1}$ and $c_{2}$), as follows:

(19) $$\xi_{c}=\sqrt{{\left(\frac{c_{1,mes}-c_{1}}{c_{1}}\right)}^{2}+{\left(\frac{c_{2,mes}-c_{2}}{c_{2}}\right)}^{2}}$$

In particular, to compare the results and the accuracy obtained from the C-LSM algorithm and the W_DIF_-LSM algorithm, the gain of efficiency function (*G*) was calculated for the W_DIF_-LSM algorithm, as follows:

(20) $$G=\frac{\xi_{C-LSM}}{\xi_{W_{DIF}-LSM}}$$

where the values $\xi_{C-LSM}$ and $\xi_{W_{DIF}-LSM}$ are the average of concentration relative error committed by two LSM algorithms. The *G* value equal to 1 represents an equal efficiency of both algorithms. When the value *G* is greater than 1, it means that the W_DIF_-LSM algorithm has better efficiency versus the C-LSM algorithm.

Moreover, the Similitude Factor ($f_{sim}$) was introduced. This value represents the percentage similitude that exists between the two synthetic spectra used to create the final spectrum and also the database. The $f_{sim}$ was calculated as follows:

(21) $$f_{sim}=1-\sqrt{\frac{\sum_{i=1}^{N}{\left(\frac{I_{1,i}-I_{2,i}}{I_{1,i}+I_{2,i}}\right)}^{2}}{N}}$$

Each result was calculated by averaging 300 tests. All tests were conducted in a range of wavelengths from 355 nm to 1024 nm with a discretization of 2050 values.

### 3.2. Results of the Numerical Tests

**Figure 1:**
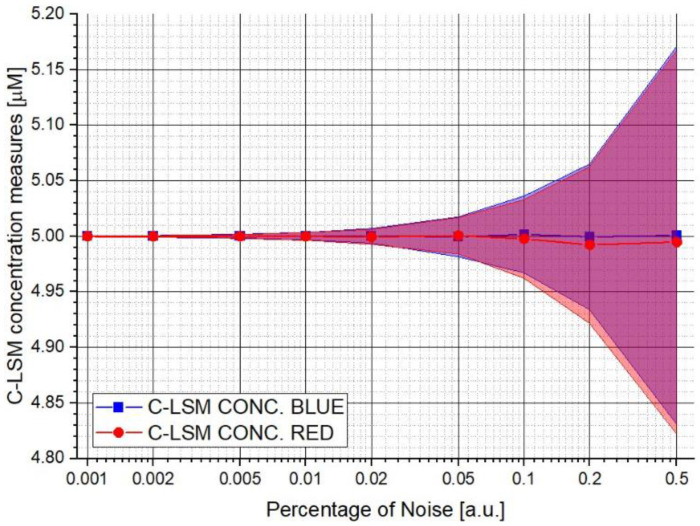
Concentration measurements made by the C-LSM algorithm varying the percentage of noise value. In the plot are reported the increment in the uncertainty in concentration measurements as a function of the increase in noise level.

Figure 1 shows the first test performed in which the measurements of the concentrations and relative uncertainty made by the C-LSM are reported as a function of the increment in the percentage noise value applied to the final synthetic spectrum. The same concentration values were imposed for the numerical tests between both hypothetical biological agents (5 μM). The couple of spectra used to generate the final spectra in this test has a Similitude Factor ($f_{sim}$) of 79.1%. It is possible to observe how the classical algorithm is influenced by the noise level. The same influence was observed for W_DIF_-LSM. It is worth mentioning that the average value does not considerably differ from the expected.

**Figure 2:**
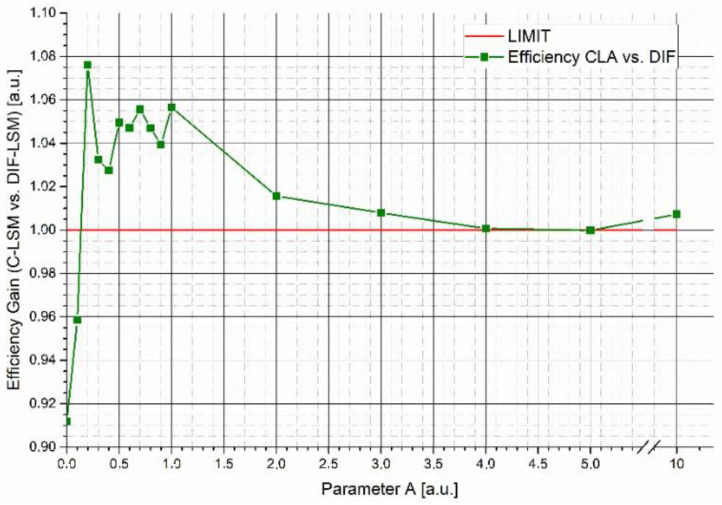
Efficiency gain (P) of the C-LSM algorithm versus the W_DIF_-LSM algorithm. In the plot are reported the trend of the efficiency gain through the two algorithms as a function of the increase in the fundamental parameter *A*.

Figure 2 shows the efficiency gain (*G*) of W_DIF_-LSM respect to C-LSM as a function of the variations in parameter *A* simulating two spectra with a $f_{sim}$ = 77.9% with the same concentrations (5 μM). The figure shows that:

1. For very small *A* parameters, W_DIF_-LSM places importance on a small number of features (only the ones where the database spectra strongly differ). This makes the inversion excessively sensitive to noise.
2. For values of *A* ranging from 0.3 to 3, the gain is greater than 1, showing that the efficiency of W_DIF_-LSM is better.
3. When parameter *A* tends to higher values (>3), the weight matrix becomes ineffective, and the results converge to the classical LSM result (*G* = 1).

Then, three different studies were conducted to examine the influence of external factors on the algorithms, such as the shape of spectra or the concentration. The first study was conducted by varying the *σ* value to change the shape of the second spectrum (taking constant the shape of the first spectrum and the concentrations). The tests were repeated sixteen times, varying parameter A every time (from 0 to 10 a.u.). The second study was conducted always changing the shape of the second spectrum, but in this case, the variable changed was $\lambda_{s}$.

**Figure 3:**
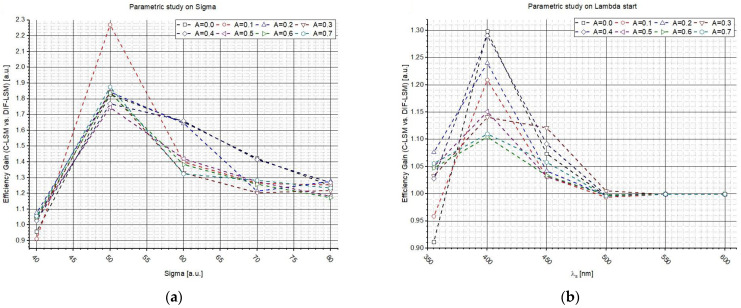

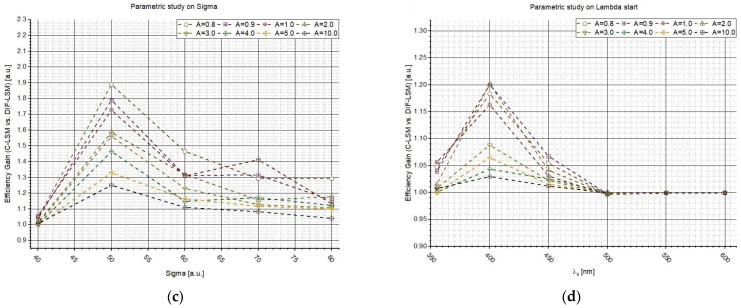
Influence of the spectrum shapes and *A* on W_DIF_-LSM varying *σ* (**a**–**c**) and $\lambda_{s}$ (**b**–**d**) of spectrum 2.

Figure 3a–c shows *G* as a function of σ, while on the right (Figure 3b–d), *G* vs. $\lambda_{s}$ is reported. The plots in Figure 3a–c clearly show that the highest gains are obtained where the spectra are similar (*σ* from 40 to 60). In fact, while the database spectra are very different, the inversion is easy to perform and a weighted LSM is not needed (even if it is worthy to highlight that the gains are always larger than one). Additionally, Figure 3b–d suggest the same conclusions: if the spectra are similar, it is relevant to use W_DIF_-LSM to increase the accuracy. For larger wavelengths, i.e., $\lambda_{s}$ > 500 nm, the spectra is so different that the inversion is almost perfect. The influence of A confirms what was observed in Figure 3.

**Figure 4:**
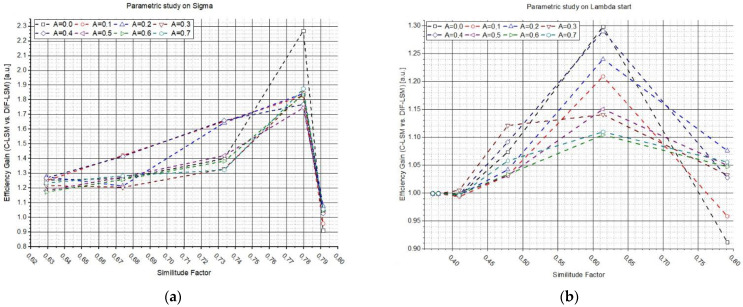

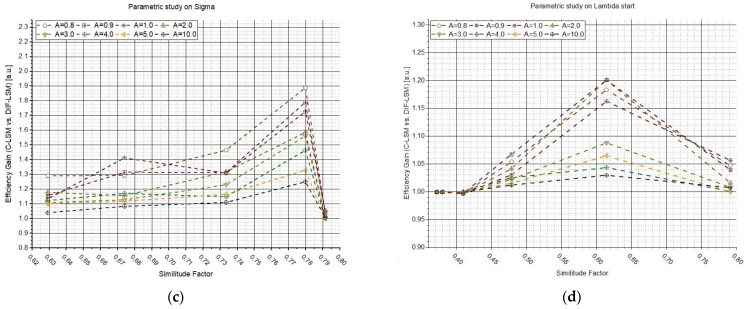
Influence of the similitude factor on the W_DIF_-LSM algorithm. Figure (**a**–**c**) shows the efficiency gain results (*G*) between the two algorithms as a function of the similitude factor ($f_{sim}$) obtained from the parametric study on *σ*. Figure (**b**–**d**) shows the efficiency gain results (*G*), for the parametric study on $\lambda_{s}$, which is reported as a function of the relative variation of the similitude factor ($f_{sim}$). Every test was conducted varying parameter *A*.

Figure 4 shows the results of the two previous studies but plotted as a function of the similitude factor between the two synthetic spectra. In plots (Figure 4a–c), the efficiency gain (*G*) values are reported as a function of $f_{sim}\;$ obtained by varying *σ*, while plots (b–d) were obtained by varying $\lambda_{s}$. Each plot line corresponds to the use of a different parameter *A* (from 0 to 10 a.u., see legend). The results confirm what was anticipated: W_DIF_-LSM allows the increase in the performances in the most challenging cases (similar spectra), while W_DIF_-LSM converges to C-LSM in the case of very dissimilar spectra.

**Figure 5:**
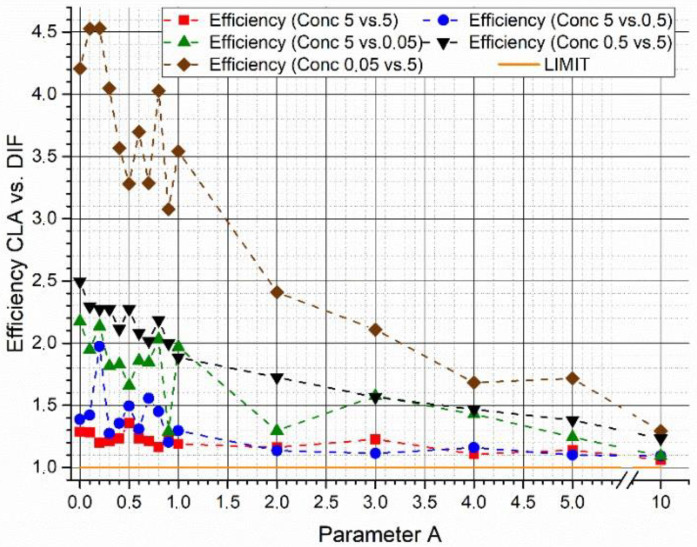
Influence of concentration differences on the efficiency gain (P) between the two algorithms. The plot describes the parametric study on concentration variations and its effect on the accuracy of the W_DIF_-LSM algorithm as a function of the parameter A variation, using for every test the same couple of synthetic spectra.

In Figure 5, the efficiency gain between the two algorithms is plotted as a function of parameter *A* as result of the parametric study at variable concentrations of the two supposed biological agents. In these tests, a constant couple of synthetic spectra with a $f_{sim}$ = 65.5% was used. The results obtained show a possible influence of concentration levels on the outcomes of the algorithms. However, although it is not possible to find a particular trend, the efficiency of the W_DIF_-LSM algorithm results in all the cases and for each value of parameter A higher than the efficiency of the C-LSM algorithm.

Finally, summarizing the results, it is possible to understand the importance of using the arbitrary parameter *A* to obtain better results from the W_DIF_-LSM algorithm. In particular, there is a range of parameter *A* values; for those values, a higher peak of efficiency in this algorithm is revealed if compared to the C-LSM one.

### 3.3. Reconstruction Error: A Quality Identifications and Measurements Indicator

To better evaluate the identification and measurements made by the algorithms, we developed a qualitative and reliability indicator before testing the algorithms on a preliminary session of experimental tests. We named this indicator as Reconstruction error and its average was calculated through the following equation:

(22) $$\eta_{rec}=\frac{1}{\sum_{i}^{n}|\Sigma \left(\lambda_{i}\right)c|}\overset{n}{\underset{i}{\sum }}|\Sigma \left(\lambda_{i}\right)c-I\left(\lambda_{i}\right)|$$

where *c* is the vector of measured concentrations, $\Sigma \left(\lambda_{i}\right)$ is the matrix spectra database and $I\left(\lambda_{i}\right)$ is the measured spectra. Through the calculation of this average value, it is possible to understand the reliability and the quality of the identification as well as for the concentration that in a hypothetically real case is unknown. Clearly, when this value is close to 100%, the classification and thus the measurements of the algorithms cannot be judged to be reliable. The necessity to develop this indicator is that the shown algorithms are characterized by the particularity that, regardless of the database being used, they always provide a result in terms of concentrations and classification, even if a detected biological agent in the environment or in the sample has not been entered in the database itself. For this reason, especially for the preliminary experimental tests, it is important to have a quality indicator for the classification and concentration measurements.

## 4. Preliminary Experimental Analyses

In this section, some preliminary experimental measurements were performed to validate the applicability of the method to real cases.

### 4.1. Materials and Methods

**Figure 6:**
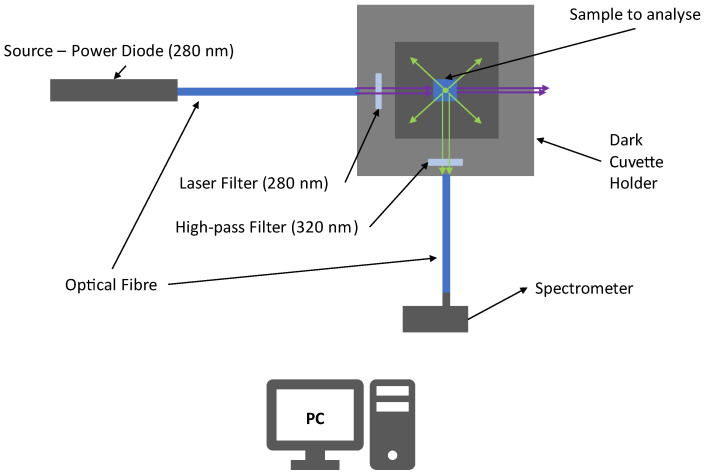
Experimental apparatus for preliminary tests of LIF classification and measurements using the supposed algorithms.

Figure 6 shows the experimental apparatus used to make the preliminary tests. The experimental apparatus is composed of a power diode source with an emission peaked around 280 nm (UV-C). The UV light is collected and transported by an optical fiber to a covered cuvette holder (in order to minimize the influence of external radiation) where the sample is located. The UV radiation is filtered by a laser filter at 280 nm (±10 nm) to obtain a quasi-monochromatic incident light beam minimizing the elastic scattering radiation that falls within the fluorescence region (*λ* > 300 nm). Then, the beam excites the sample and Laser-Induced Fluorescence radiation is stimulated. A gap in the cuvette older allows the collection of UV light emitted at 90°. The such emitted light is filtered with a high-pass filter that stops radiation at λ < 320 nm, decreasing the magnitude of elastic scattering radiation.

Then, the fluorescence signals are finally collected and transported to the spectrometer.

The spectrometer used for this analysis was a QePro-UV-VIS, by Ocean Optics, which has a cooled-CCD with a wavelength range from 197 nm to 792 nm, with 1044 pixels and a resolution of about 0.57 nm, while the inlet slit to collect the signals has a diameter of 200 μm to collect the biggest amount of fluorescence.

All spectra acquired for the preliminary tests were collected from wavelengths ranging from 364 nm to 792 nm.

To perform the preliminary tests of the algorithms in the experimental condition, different samples of mixtures of Riboflavin (RB) and *Bacillus clausii* spores (BC) as a positive control were prepared dissolving the agents in purified water and mixing them. Two different solutions were prepared as stock solutions:

- BC spore solution with a concentration of 0.8 × 10^9^ spores per mL;
- RF solution with a concentration of 2.6 × 10^−4^ M.

The database of two spectra was built to measure the fluorescence of the two components with different concentrations with the same exposure time of 5 s, for ten times per sample:

- For the RF database spectrum, the fluorescence spectra were acquired at two different concentrations: 1.33 × 10^−7^ M and 2.66 × 10^−8^ M.
- For the BC database spectrum, the fluorescence spectra were acquired at two different concentrations: 0.04 × 10^9^ spores per mL and 0.008 × 10^9^ spores per mL.

**Figure 7:**
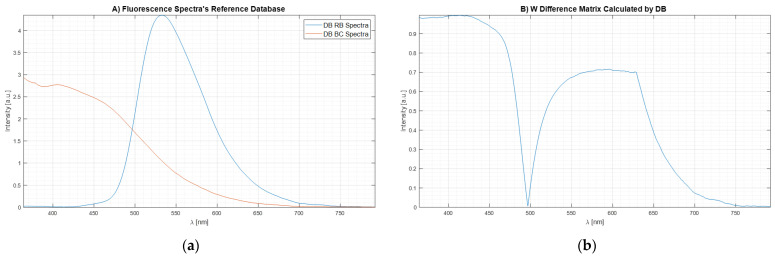
Database spectra of the two agents (**a**); weight matrix for W_DIF_-LSM (**b**).

Ten spectra per sample were acquired and the reference (or database) spectra were obtained by averaging them and by removing the background (residual scattering in pure water). Then, the spectra were normalized by the concentration and the exposure time:

(23) $$\rho_{fl,j}\left(\lambda_{i}\right)=\frac{I_{j}\left(\lambda_{i}\right)}{c_{j}*t_{exp}}$$

where $I_{j}$($\lambda_{i}$) is the fluorescence intensity measured in $c_{j}$ concentration condition with a time of exposure $t_{exp}$. Then, the two resulting spectra per agent were averaged. These spectra are the ones that compound the database (Σ). The results are shown in Figure 7a.

Then, the $W_{dif}$ matrix was calculated according to the previous sections. The equation is reported here for the reader’s convenience:

(24) $$W_{Dif}=\left|\frac{\rho_{BC}-\rho_{RF}}{\max\left(\rho_{BC}+\rho_{RF},1\right)}\right|$$

Figure 7b shows the plot of the weight matrix for W_DIF_-LSM obtained through Equation 24 using the spectrum data reported in Figure 7a. It is important to see how, when the two signals intersect having the same intensity (λ<500 nm, Figure 7a), the same point in the weight matrix (Figure 7b) is equal to 0 because there are not differences between the two Bas spectra. At the same time, when the spectra signals are equal to 0 (λ>750 nm, Figure 7a), the values of weight matrix (Figure 7b) are equal to 0 because there are no differences.

The A parameters (see Equation (14)) tested were 21 values ranging from 0 to 10 with a step size of 0.5.

The data of the mixture samples used for these preliminary experimental tests are reported in Table 1. 

Ten measurements for each sample were executed to obtain the average fluorescence spectra of each sample that were filtered by the average background spectrum, and finally normalized by the exposure time of 5 s before the analysis through the two LSM algorithms.

### 4.2. Results of the Preliminary Experimental Tests

**Figure 8:**
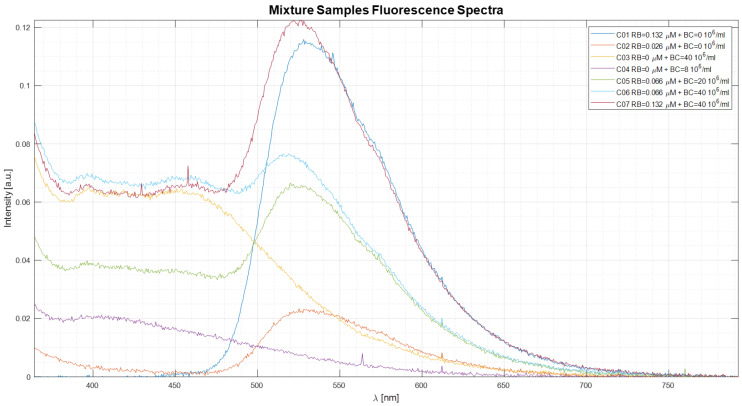
The plot shows the average fluorescence spectra of the mixture samples.

Figure 8 shows the average exposure time normalized fluorescence spectra obtained by the 10 spectra made for each mixture sample. Before the normalization, each average spectrum was cleaned from the background spectrum.

In all experimental cases, the average reconstruction error $\eta_{rec}$ was smaller than 6% for both algorithms. 

**Figure 9:**
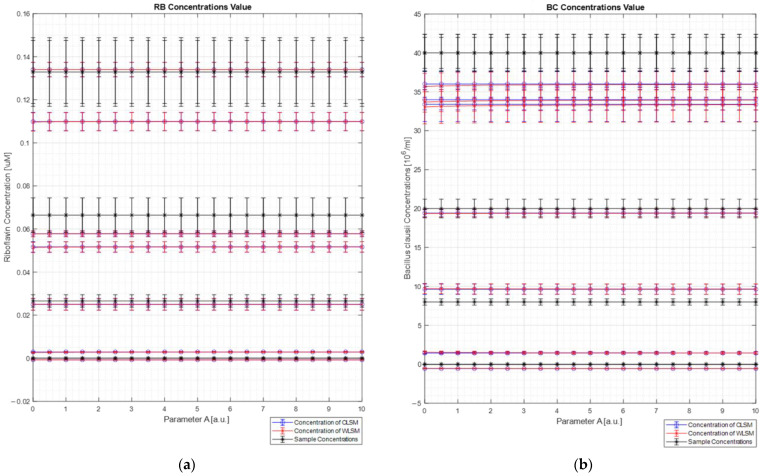
Concentration measurement results for each biological agent (Riboflavin (**a**) and *Bacillus clausii* (**b**)) for each sample. In particular, in each plot, the expected concentrations of each biological agent in each sample (black star) are plotted with its bar of uncertainty value related to the dilution and mixture preparation. The concentrations of each agent calculated by C-LSM are reported in blue, while the concentrations of each agent calculated by W_DIF_-LSM are shown in red. Each calculated concentration value is indicated with its error bar, which is the uncertainty of the value calculated as the product between the reconstruction error and the concentration calculated by the algorithms.

Figure 9 shows the concentration results obtained for each sample (Figure 9a for the Riboflavin concentration values, and Figure 9b for the *Bacillus clausii* concentration values) of both the algorithms (blue line for the C-LSM results and red line for the W_DIF_-LSM results) in comparison with the expected concentration (black line in each plot) as a function of parameter A. For each concentration’s value, the uncertainty measurements calculated through the uncertainty propagation theory are reported [13]. In particular, the relative error on concentration measurements made by both algorithms (Equation (19)) is between 13% and 36%. These large errors are mostly due to two different limits. First, a small portion of elastic scattering is still present, and it makes the reconstruction not perfect (error ranging from 2% to 10%). Moreover, the spectrometer has a nonlinear response (a doubling of the intensity does not involve a doubling of the measured counts). Therefore, these two factors reduced the quality of the reconstruction and the extrapolation of the concentrations.

As can be observed from the figure, both the LSM techniques return the same results (considering the relative error bars). This result was expected since the database spectra are very dissimilar, and it makes the weight spectrum not very informative (see Figure 7b). This result confirms what was analyzed in the numerical section, where it was clearly observed that, for dissimilar spectra, W_DIF_-LSM converges to C-LSM.

## 5. Conclusions

In this work, a new weighted-LSM method was developed to increase the accuracy of the classification and concentration measurements from LIF spectra. These two algorithms can be applied supposing a linear dependence between the concentration and the intensity of fluorescence spectra. It is known that this phenomenon of linearity is true only for low concentrations of biological agents in the sample.

Starting from the LSM method, the weighted approach was developed and refined to ask the algorithm to weigh more regions where agent-specific features are observed.

The capabilities and the accuracy of the new algorithm were tested through two different approaches: numerically, using a synthetic spectrum generated through mathematical functions, and experimentally, by performing measurements with a LIF apparatus on two bio-agents.

The numerical tests allowed us to obtain an initial perspective on the capabilities of the two algorithms (C-LSM and W_DIF_-LSM) and their behavior simulating the conditions in which the algorithms must operate. As expected, it was possible to observe the presence of an influence represented by the background noise on the tested algorithm. 

Through the simulation, testing W_DIF_-LSM, it is possible to see how the efficiency of this algorithm is higher than the efficiency of C-LSM. In particular, it is possible to see how the regularization of parameter A plays an important role in the algorithm’s performance. For very small values of A, W_DIF_-LSM does not place importance on many wavelengths. The information used to extract the concentration decreases and thus the uncertainties increase. Increasing the A values, the algorithm finds a balance between “weighting all intensities” and “weighting only the most relevant” and the inversion performances increases significantly. For very large A values, W-LSM converges to C-LSM. 

Moreover, the similitude effect of the two database spectra was analyzed and it was observed that the new algorithm, W_DIF_-LSM, is able to return higher gains when the problem is more complex, i.e., when the two spectra are more similar. This is of course due to the fact that, when the spectra are strongly different, C-LSM obtains very high performances, which is probably impossible to improve.

In the case of real applications, the expected concentrations are not known. Therefore, in order to have an indicator able to provide an idea of the inversion reliability, the reconstruction error indicator was developed. This indicator returns a value proportional to the difference between the measured and the reconstructed spectrum. When its value is small, it means that the combination of the database spectra with the measured concentrations allows us to reconstruct the measured spectrum correctly, suggesting that the two concentrations should be close to the actual value. 

The preliminary experimental tests were not as good as the numerical ones. However, these outcomes depend on some experimental limits. At first, the authors used biological agents with very dissimilar spectra, where the new W_DIF_-LSM algorithm did not have appreciable advantages (demonstrated also by the numerical analysis). Secondly, the spectrometer has a non-linear uncalibrated response that makes the spectra counts not directly proportional to the concentration of the agents. At last, a small amount of scattering radiation was still present, making the spectra harder to analyze. However, the last two points are typical problems in real applications that can not always be excluded. Therefore, it is important to note that, in such unfavorable conditions, even if the results are not perfect, the concentration estimation is correlated to the expected ones and that no absurd results were obtained (see Figure 9). 

It must be highlighted that the present algorithm, contrary to supervised machine learning algorithm such as regression trees and neural networks, does not need supervised training with several samples, involving a direct application. 

In conclusion, the authors think that this new approach may be applied to those conditions where reference spectra are very similar (see, for example, the study conducted by Duschek et al. [14]). In such cases, W_DIF_-LSM may truly increase the performance of the concentration extrapolation, allowing more reliable and accurate results. 

Despite this new method being presented for LIF spectra, it is worth mentioning that it may be applied to several other spectroscopic measurements that share an equation similar to the one presented in Equation (3), such as absorption spectroscopy and laser-induced breakdown spectroscopy (in certain situations, of course).

## Figures and Tables

**Table 1:** Sample composition used for experimental tests and exposure time used for each acquisition.

Samples	RF Concentration [μM]	BC Concentration [10^6^ Spores/mL]	Exposure Time [s]
Sample C01	0.132	0	5
Sample C02	0.026	0	5
Sample C03	0	40	5
Sample C04	0	8	5
Sample C05	0.066	20	5
Sample C06	0.066	40	5
Sample C07	0.132	40	5

## Data Availability

The data presented in this study are available on request from the corresponding author.
